# Evaluation of surface water quality indices and ecological risk assessment for heavy metals in scrap yard neighbourhood

**DOI:** 10.1186/s40064-016-2158-9

**Published:** 2016-05-04

**Authors:** Olusheyi Z. Ojekunle, Olurotimi V. Ojekunle, Azeem A. Adeyemi, Abayomi G. Taiwo, Opeyemi R. Sangowusi, Adewale M. Taiwo, Adetoun A. Adekitan

**Affiliations:** Federal University of Agriculture, Abeokuta, Ogun State Nigeria; Moshood Abiola Polytechnic, Abeokuta, Ogun State Nigeria; State Key Laboratory of Geomechanics and Geotechnical Engineering, Chinese Academic of Sciences, Wuhan, China

**Keywords:** Heavy Metal Pollution Index, Metal Index, Potential Ecological Risk Index, Scrap yard and surface water

## Abstract

Pollution of surface water with heavy metals from industrial activities especially those from scrap yard has caused a major threat to human life exposing man to series of hazard, diseases, disability and consequently death. This study focuses on water quality indices of Owode-Onirin and Lafenwa scrap yard with respect to its physicochemical parameters and heavy metal concentrations by evaluating Heavy Metal Pollution Index (HPI), Metal Index (MI) and Potential Ecological Risk Index (PERI). Fifteen water samples were selected randomly from two locations by purposive sampling methods. Five heavy metals which includes Nickel (Ni), Zinc (Zn), Copper (Cu), Cadmium (Cd), Lead (Pb) were analyzed using Atomic Absorption Spectroscopy and standard analytical procedure were follow to ensure accuracy. One way analysis of variance was carried out to analyse the data. The concentrations of the heavy metals were significantly different between sampling locations. However, the mean concentrations of Cd (0.0121 mg/L) were found to be above the highest permissible value of Standard Organization of Nigeria standards for drinking water (SON 2007) and WHO (Guidelines for drinking water quality: incorporating 1st and 2nd Addlenda. World Health Organization, Geneva, 2004) for drinking water. Although Pb was present in two out of the fifteen water samples with a mean value of (0.0324 mg/L) which was also above the highest permissible value. The mean concentrations of Zn (0.2149 mg/L) and Cu (0.0341 mg/L) are found to be below the highest permissible value of the mentioned guideline while no trace of Ni was found in the water samples across the two sampling locations. The mean HPI 518.55 is far above the critical value of 100, indicates that selected water samples are critically polluted with heavy metals. MI revealed low quality water with mean value 4.83, suggests that the selected water is seriously affected with the present of heavy metal. The Hakanson PERI indicated that of the five heavy metals, the risk coefficient of Zn, Pb, Cu, and Ni had light levels of contamination while the level of Cd contamination posed the most serious potential ecological risk, with an index value between 14.1 and 234. The study concluded that order of magnitude to this five heavy metals contamination is Cd > Pb > Zn > Cu > Ni.

## Background

Water is an important component of rural and urban environments and management of this proponent is the key to ensuring a better and quality environment. However, the generation of different types of wastes such as motor parts, scrap of cars, engines, cans tyres, etc., are a result of globalization in many parts of world and the developing countries in particular. The waste produced known as scraps are often dumped in areas where heavy metals and other components are accessible to leaching and causing havoc to the ecosystem.

Weiss (1974), observe that pollution of groundwater impaired water quality necessarily which result in public health hazards, Ogbonna et al. (2006) further stressed that it will adversely affect such water for domestic, farm, industrial and municipal use (Akhilesh et al. 2009).

Investigation of heavy metals is very essential according to Yahaya et al. (2009), since little modification in their concentration above the threshold levels to biogenic or anthropogenic factors, leading to serious environmental hazard and subsequent welfare health problems. Klavins et al. (2000), Tam and Wong (2000), Yuan et al. (2004), Hakan (2006) all reiterated that heavy metals are serious environmental pollutants with toxicity tendency, longevity and persistency in the environment. Environmental pollution by heavy metal ions arises as a result of many activities in the environment. In the soil system, pollution by toxic metals is due to both biogenic processes (weathering of minerals) and anthropogenic activities (agriculture, burning of fossil fuels, industry, scrap yards, vehicular emissions, mining and metallurgical processes and waste disposal) as investigated by Kumar (2005), Biasioli et al. (2006), and Martin et al. (1982) while concluding that heavy metal contamination in the soil–water–plant ecosystem is of great importance because of possible influence on food chain (Gray et al. 2003).

Heavy metal contaminants could be chemical and biological processes in nature with a potential impact on human health and environmental welfare (Giuliano et al. 2007). The presence of heavy metals in and around urban areas has been an area of great concern due to their long persistent nature and long biological half-lives within the human system when taken. Negative effects due to heavy metal contamination in surface and underground water are well established by Tumuklu et al. (2007) for Manganese, Chromium and Zinc causing neurosis and chlorosis while Nickel, Cobalt and Cadmium hinder stomata activity and decrease photosynthesis in plants (Prasad 1995). Aluminum, Cobalt, Copper, Iron, Lead, Manganese, Nickel and Zinc were reported to cause potential hazards in water (Grigalaviciene et al. 2005; Tumuklu et al. 2007; Al-Kashman and Shawabkeh 2009).

Akoto et al. (2008) in his finding documented that in most developing countries, scrap yards are increasing and will continue to expand from rapid economic growth through population increase, industrialization, and increased motorization. Although heavy metals distribution and concentration in soluble water is well documented for a number of places of developed countries, there is a scarcity of information from less developed countries.

## Review/Background

Al-Weher (2008) investigated the contamination of water by heavy metal but their levels have expanded due to some of the following which includes agricultural, mining, domestic, industrial activities etc. (Kalay and Canli 2000; Yousalfzai and Shakoori 2008). The increase concentration of these elements beyond the levels required can be toxic as investigated by Gulfaraz et al. (2001).

Industrial pollution seriously posed a problem to the quality of water resources and the environment at large. Refuse from food industries has been reported in defecating water from almost all the boreholes around Bompai industrial area (Egboka et al. 1989). Tanko (2002) investigated the incidence of water discharge as arguably the most threat to city farming and environmental hazard in the region. A study also conducted in 1989, which examined the activities of 15 tanneries in northern part of Kano State (World Bank 1995), investigated that in all cases, acceptable limits for effluents discharged were not adhered to, so also was downstream fish and crops affected heavy metals pollution, hence human health in Kano is greatly threatened, since over 60 % of the local people relied on ground and surface water for their household use.

Better understanding of qualitative water and ecological status of most studied systems are often done through the application of various multivariate approach for interpretation of data matrices, its allow for identification of possible sources/factors that affect water systems and provide a variable tool for dependable management of both ground and surface water resources as well as solution to tackle pollution problems The study of groundwater circulation system in Swietokrzyski National Park was carried by Krishna et al. (2009), Michalik (2007) while applying statistical analysis. It also used it to trace similarities or differences between sampling sites. Guash et al. (2009) deployed multivariate statistical analysis to evaluate the relative contribution of different types of pollution to diatomic species.

The study of Stiles et al. (2004) applied statistical tools to assess mine water quality and to reveal the hydro geochemical parameter/constituents of River Povpov in Itakpe iron-ore mining Area, Kogi State, Nigeria and that was also reported by Sekabira et al. (2010). This study tends to advance the course of knowledge by utilizing some relevant water related index to ascertain the level of contamination of heavy metal on surface water in Nigeria.

## Study area

**Fig. 1:**
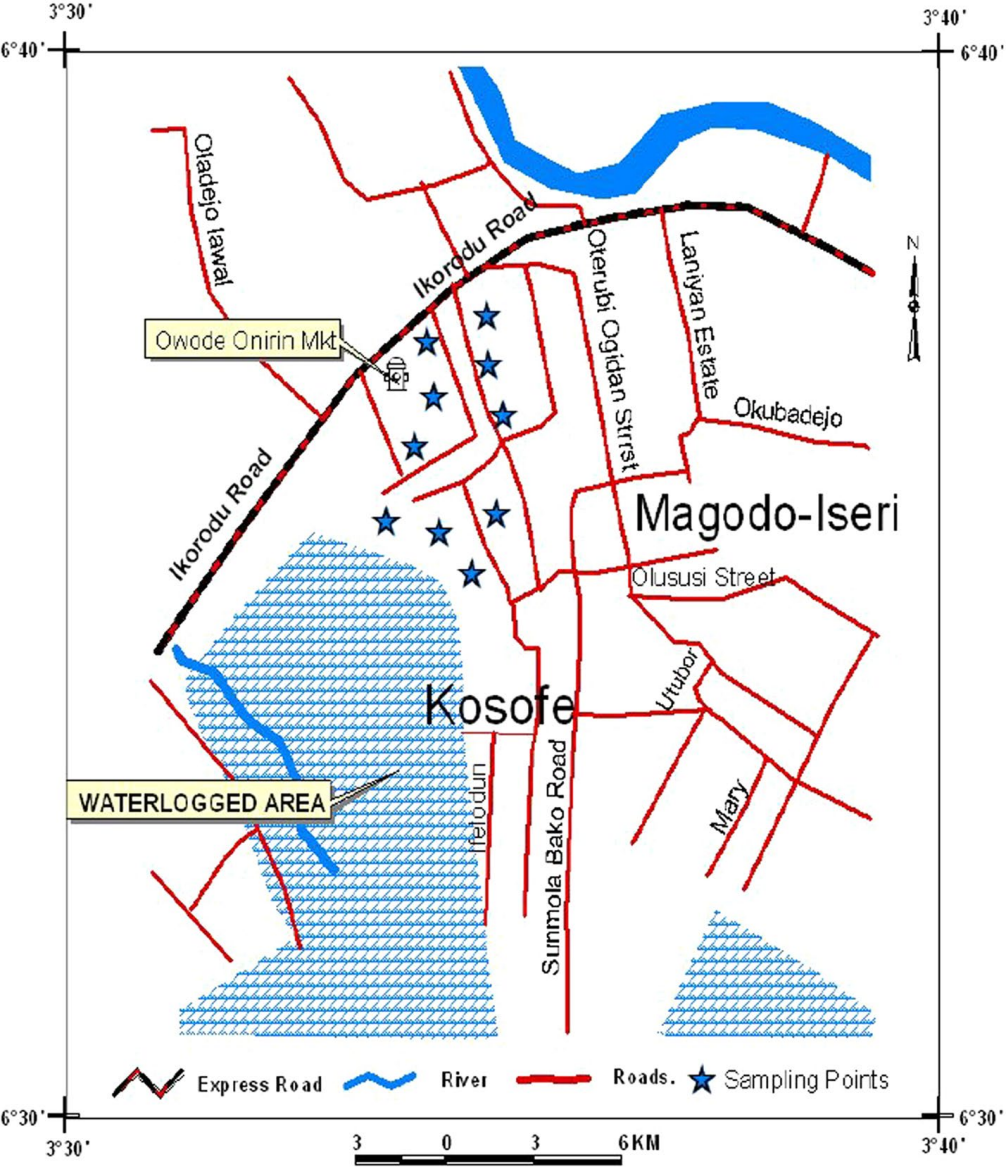
Owode-Onirin, Ikorodu area of Lagos State *Source* GIS Mapping (FUNAAB, 2014)

The study area at Owode-Onirin, Ikorodu area of Lagos State, Nigeria which lies between latitudes 6°36′14.87′′N and 6°36′32.4′′N and longitudes 3°24′47.05′′E and 3°24′48.6′′E (see Fig. 1) is said to have been established over 30 years ago. It is divided into two sections: metal fabrication and automobile section. While the scrap section of the market is home to different kinds of scraps one can ever imagine, the vehicle section is particularly known as the zone of used cars, including tokunbos and third-hand vehicles. Owode-Onirin, Ikorodu area of Lagos State is well known for the dumping of these scraps that degrades the environment and affects the scenery of the areas. Little attention had been given to the maintenance of the scrap yard over the years making the public around the neighborhood vulnerable to these heavy metals leaching especially to surface water.

**Fig. 2:**
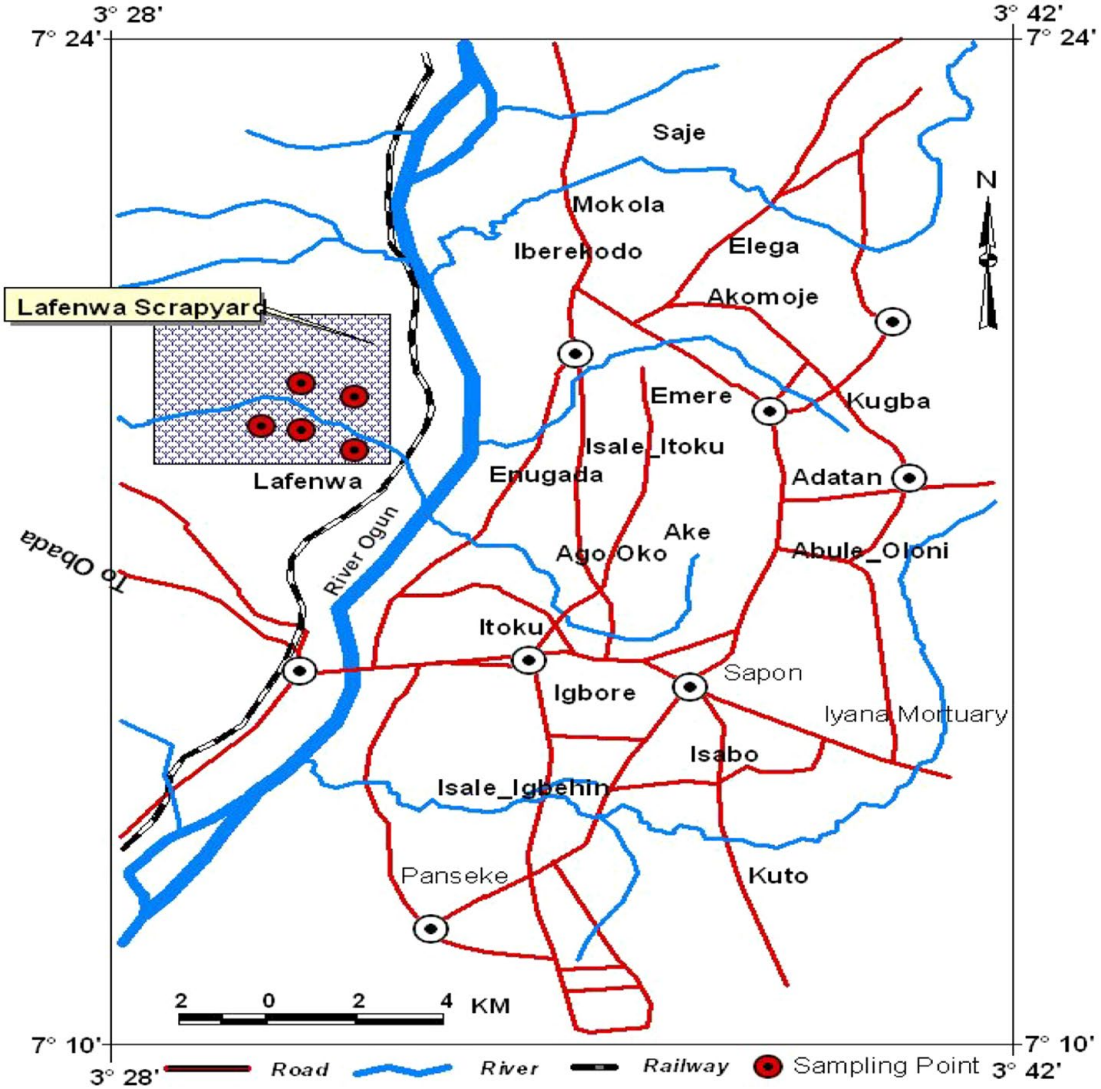
Map showing the sampling locations in Lafenwa, Abeokuta area of Ogun State *Source* GIS Mapping (FUNAAB, 2014)

The study area Lafenwa scrap yard, Abeokuta is located in the sub-humid tropical region of Southwestern Nigeria between latitudes 7°9′18.86′′ N to 7°9′19.62′′ N and longitudes 3°19′27.48′′ E to 3°19′28.31′′E (see Fig. 2). The town is about 81 km south-west of Ibadan and 106 km North of Lagos and at an altitude of about 157 m above sea level, the landscape has undulating characteristics due to the formation of granite rocks. The city as studied by Orebiyi et al. 2007 enjoys a tropical climate with two distinct seasons (rain and dry) with the dry length of about 4–5 months. He also investigated that the average rainfall and temperature per annum are about 1270 mm and 28 °C respectively while the estimated average yearly potential evaporation is 1100 mm. The city is underlain by crystalline pre-Cambrian Basement complex of igneous and metamorphic origin noted for poor groundwater bearing properties. The city is drained mainly by the Ogun River which divides the city into two, and the drainage pattern is dendritic in nature. The study area which covers a geographical area of 1256 km^2^ has a population of about 610,000 and comprise of Abeokuta South, Abeokuta North, parts of Odeda and Obafemi-Owode Local Governments of Ogun State of Nigeria. The main occupation of the indigenes is local textile making (Adire) trading, fishing, farming and pottery.

## Methods

### Sample collection and processing

A total number of 15 samples were collected over some period of time across the two distinct study location (Owode-Onirin, Ikorodu area of Lagos State and Lafenwa scrap yard, Abeokuta area of Ogun State) using 1 L plastic containers for the collection following standard procedures. Water sample was collected in a hand dug well sited within the scrap yard area of Owode-Onirin, Lagos State and Lafenwa scrap yard, Abeokuta area of Ogun State and also in a nearby stream around Lafenwa scrap yard in Abeokuta, Ogun State as shown on Table 1. The water appearance, pH, temperature, Total dissolved solids and Electrical conductivity of the water samples were determined in situ. Prior to sample collection, precautions were taken to ensure that the samples to be collected are free from contamination.

**Table 1:** Location of sampling points. *Source* Author’s Field Work 2014

S/n	Location	Sample code	Sample type	Latitude	Longitude
1.	Owode-Onirin	ODS-1	Hand dug well	N. 6.60413	E. 3.41307
2.	Owode-Onirin	ODS-2	Hand dug well	N. 6.60691	E. 3.41332
3.	Owode-Onirin	ODS-3	Hand dug well	N. 6.60757	E. 3.41225
4.	Owode-Onirin	ODS-4	Hand dug well	N. 6.60805	E. 3.4123
5.	Owode-Onirin	ODS-5	Hand dug well	N. 6.6087	E. 3.41239
6.	Owode-Onirin	ODS-6	Hand dug well	N. 6.60844	E. 3.41283
7.	Owode-Onirin	ODS-7	Hand dug well	N. 6.6088	E. 3.4134
8.	Owode-Onirin	ODS-8	Hand dug well	N. 6.605	E. 3.41345
9.	Owode-Onirin	ODS-9	Hand dug well	N. 6.608	E. 3.41348
10.	Owode-Onirin	ODS-10	Hand dug well	N. 6.609	E. 3.41350
11.	Lafenwa	LFS-1	Stream	N. 7.15545	E. 3.32453
12.	Lafenwa	LFS-2	Stream	N. 7.15544	E. 3.32454
13.	Lafenwa	LFS-3	Stream	N. 7.15529	E. 3.32444
14.	Lafenwa	LFS-4	Hand dug well	N. 7.15524	E. 3.3243
15.	Lafenwa	LFS-5	Hand dug well	N. 7.15527	E. 3.32421

### Determination of physicochemical parameters

This entails analysis of the physical properties as well as the chemical content of water samples. Total alkalinity was determined by the hydrochloric acid titrimetric method (methyl orange), total chloride was determined by silver nitrate titrimetric method (potassium chromate), total hardness was determined by ethylenediamine tetra acetic acid (EDTA) titrimetric method (Erichrome black T), pH was determined by electrode meter, TDS was determined by electrode meter, EC was determined by electrode meter, Temperature was determined by electrode meter while Nitrate, Sulphate, Phosphate, Sodium and Potassium were determined by spectrophotometric method. The results of physicochemical parameters were shown in Table 2.

**Table 2:** Physical and chemical characteristics of water sampled *Source* Author’s Field Work 2014

Sample code	pH	Temp (°C)	EC (μS/cm)	TDS (mg/L)	Alkalinity (mg/L)	Chloride (mg/L)	Hardness (mg/L)	PO_4_^3−^ (mg/L)	SO_4_^2−^ (mg/L)	NO_3_^−^ (mg/L)	Na^+^ (mg/L)	K^+^ (mg/L)
ODS-1	9.61 ± 0.03	29	1060	530	10	93	266	0.051	83.7	14.331	19	5
ODS-2	9.66 ± 0.06	30	1230	615	13	139	278	0.071	77.7	14.966	25	9
ODS-3	9.92 ± 0.03	29	900	450	9	64	268	0.010	63.9	14.150	17	7
ODS-4	9.84 ± 0.05	29	700	350	11	59	248	0.061	54.19	12.608	17	6
ODS-5	9.77 ± 0.02	30	782	391	7	66	196	0.051	94.7	12.517	18	5
ODS-6	9.54 ± 0.04	29	800	400	8	69	190	0.051	76.3	13.787	17	4
ODS-7	9.95 ± 0.01	29	800	400	10	78	294	0.061	58.2	7.438	17	8
ODS-8	10.20 ± 0.02	29	1378	689	13	157	314	0.162	51.5	21.406	24	6
ODS-9	9.87 ± 0.02	29	552	276	9	65	144	0.122	21.1	19.773	17	3
ODS-10	10.07 ± 0.01	31	456	228	10	56	140	0.122	21.0	22.766	14	2
LFS-1	10.31 ± 0.03	28	474	237	8	44	154	0.456	28.9	29.297	9	3
LFS-2	10.36 ± 0.03	28	500	250	12	40	114	0.901	27.7	32.653	11	4
LFS-3	9.64 ± 0.02	30	980	490	13	410	500	0.172	30.6	58.594	90	100
LFS-4	9.81 ± 0.06	29	500	250	12	386	600	0.122	57.9	58.957	90	60
LFS-5	9.75 ± 0.02	29	460	230	11	685	450	0.122	54.8	55.782	90	60

### Analysis and determination of heavy metals

100 mLs of water sample was measured using a measuring cylinder and was placed into a digestion vessel. 10 mLs of concentrated hydrochloric acid was added to the water sample in the digestion vessel and placed on the heating source. The sample was heated for about 20–25 min for complete digestion after which the digest was allowed to cool for about 24 h before the digest was made up to 100 mLs in a standard flask and stored for elemental analysis. A blank sample was also prepared. Pb, Cu, Zn, Ni and Cd were determined by Atomic Absorption Spectrophotometry. The distribution characteristics of heavy metals in the study area are shown in Table 3.

**Table 3:** The results of heavy metals concentration of the sample water vis-à-vis the regulatory standards limits of World Health Organization and Standard Organization of Nigeria (World Health Organization 2006, 2008)

Sample code	Zn (mg/L) WHO 3 mg/L SON 3 mg/L	Cu (mg/L) WHO 1 mg/L SON 1 mg/L	Cd (mg/L) WHO 0.003 mg/L SON 0.003 mg/L	Pb (mg/L) WHO 0.01 mg/L SON 0.01 mg/L	Ni (mg/L) WHO 0.02 mg/L SON 0.02 mg/L
ODS-1	0.0748	ND	0.0193	0.0188	ND
ODS-2	0.0713	ND	0.0147	ND	ND
ODS-3	0.1615	ND	0.0139	ND	ND
ODS-4	0.0790	ND	0.0147	ND	ND
ODS-5	0.0778	ND	0.0131	0.0459	ND
ODS-6	0.1258	ND	0.0086	ND	ND
ODS-7	0.0899	ND	0.0064	ND	ND
ODS-8	0.0479	ND	0.0100	ND	ND
ODS-9	0.0532	ND	0.0051	ND	ND
ODS-10	0.0470	ND	0.0110	ND	ND
LFS-1	0.0762	ND	0.0014	ND	ND
LFS-2	0.0962	ND	0.0193	ND	ND
LFS-3	0.7204	0.0341	0.0147	ND	ND
LFS-4	0.7781	ND	0.0139	ND	ND
LFS-5	0.7239	ND	0.0147	ND	ND

## Discussion of results

### Physical and chemical results

Mean pH of the Owode-Onirin scrap yard and Lafenwa scrap yard neighbourhood for all water samples were (9.54–10.20) which are above the permissible limit range of 6.5–8.5 specified by WHO (2011). According to the pH values obtained, majority were in the trend of slightly alkaline. Therefore, the water samples were unlikely to cause health problems such as acidosis (Asamoah and Amorin 2011). However, pH played a significant role in determining the bacterial population growth and diversity in water body. Increases in the observed pH, could be attributed to the production of basic metabolic waste products by increasing bacterial population and reduces availability of heavy metals (Ciavatti et al. 1993). In their review, Prescott et al. (1999) stated that microorganisms frequently change the pH of their own habitat by producing acidic or basic metabolic waste products.

The temperature readings of the sachet water samples are presented above. The values ranged from 29.0 to 31.0 °C (ODS) and 29.0 to 30.0 °C (LFS). The temperature values obtained throughout the investigation period fall within the optimal growth range for mesophilic bacteria including human pathogens. Prescott et al. (1999) reported 20–45 °C as optimal growth temperature for mesophilic micro organisms. According to WHO report (1996), the microbiological characteristics of drinking water are related to temperature through its effects on water-treatment processes and its effects on both growth and survival of micro organisms. Consequently, growth of micro organisms is enhanced by warm water conditions and could lead to the development of unpleasant tastes and odours.

Conductivity values express the amount of dissolved solids in the water sample. Water from Owode-Onirin scrap yard and Lafenwa scrap yard neighbourhood has conductivity values that ranged from 456 to 1378 and 460 to 980 μS/cm respectively. Conductivity of the water samples was above the stipulated value of 1000 μS/cm by WHO and NAFDAC. Although may be high with attended increase in value over time. The observed data of electrical conductivity followed similar pattern as pH. It was observed that EC value increased in the contaminated water and this revealed the positive impact of scrap yard on contaminated water EC. High EC can affect the microbial activities in soil (USDA-NRCS 2014).

The total suspended solid was found to ranged from 228 to 689 and 237 to 490 mg/L for Owode-Onirin scrap yard and Lafenwa scrap yard neighbourhood respectively with Owode-Onirin scrap yard higher than Lafenwa scrap yard neighbourhood. While a direct correlation can be established in Owode-Onirin scrap 8 and 2 for Chloride (157 and 139 mg/L) and EC with (1378 and 1230 μS/cm) respectively which high chloride values for Lafenwa scrap yard neighbourhood do not show these trend hence the variation in the mean values varied significantly amongst the samples as the values obtained were different which may be attributed to further infiltration of salt along the coastal line. The values of hardness also show a direct relationship with the concentration of Chloride with the same Owode-Onirin scrap yard having higher concentration, Chloride ions are non-cumulative toxins, an excess amount of which, if taken over a period of time, can constitute a health hazard. It is believed that higher concentration of chloride ions may result in taste problems and higher level of chloride is known to impart taste to water particularly when sodium is the predominant cation (APHA 2005). Though, stronger relationship is exhibited in the values of chloride and hardness in Lafenwa scrap yard neighbourhood 5, 3 and 4 in that order of magnitude and the values were all above the WHO (2011) standard. Nitrate and potassium were also found to be higher in the same three site of the Lafenwa scrap yard neighbourhood well above the WHO (2011) stipulated standard, increases in nitrate values could be attributed to their utilization by micro organisms for growth and reproduction along the scrap yard (Prescott et al. 1999), high level of sodium can also lead to a high Na/K and Na/total cation ratio which may be of great concern from the perspective of human pathology (NRC 1989; APHA 2005). Although the increased in sodium other micro nutrient fall within the permissible limit and hence posses no threat to the environment.

### Heavy metals results

The results of the concentrations of the five studied heavy metals have been shown in Table 4. The metal concentrations were significantly different between sampling locations. However, the mean concentrations of Cd (0.0121 mg/L) were found to be above the highest permissible value (0.003 mg/L) of Standard Organization of Nigeria standards for drinking water (SON 2007). However Pb was present in two of the fifteen water samples with a mean value of (0.0324 mg/L) which was also above the highest permissible value of (0.01 mg/L). The concentration of Ni was not detected at any level after drawing blanks of the samples analysed, while the mean concentrations of Zn (0.2149 mg/L) and Cu (0.0341 mg/L) are found to be below the highest permissible value of the mentioned guide line of 3 and 1 mg/L respectively which has a possibility of been from Galvanised Roofing Sheet and Ceramic industrial wastes. Based on the concentration ranges and abundance, heavy metals are ranked in order of magnitude as Cd > Pb > Zn > Cu > Ni in Owode-Onirin scrap yard and Lafenwa scrap yard neighbourhood.

**Table 4:** Water quality classification using Metal Index (MI). *Sources* Lyulko et al. (2001); Caerio et al. (2005)

MI	Characteristics	Class
<0.3	Very pure	I
0.3–1.0	Pure	II
1.0–2.0	Slightly affected	III
2.0–4.0	Moderately affected	IV
4.0–6.0	Strongly affected	V
>6.0	Seriously affected	VI

Heavy Metal Pollution Index (HPI) is a technique of rating that provides the aggregate influence of individual heavy metal on the total water quality. The rating is a value between 0 and 1, reflecting the relative/subjective importance of each quality considerations and inversely proportional to the recommended standard (Si) for each parameter (Reza and Singh 2010; Prasad and Mondal 2008; Prasad and Sangita 2008). The calculation of HPI involves the following steps:

- First, the calculation of weightage of ith parameter.
- Second, the calculation of the quality rating for each of the heavy metal.
- Third, the summation of these sub-indices in the overall index.

The weightage of ith parameter is given by

$$W_{i} = \frac{k}{{S_{i} }}$$

where W_i_ is the unit weightage and S_i_ the recommended standard for ith parameter, while k is the constant of proportionality.

Individual quality rating is given by the expression below

$$Q_{i} = \frac{{100 \times V_{i} }}{{S_{i} }}$$

where Q_i_ is the sub index of ith parameter, V_i_ is the monitored value of the ith parameter and S_i_ the standard or permissible limit for the ith parameter.

The Heavy Metal Index (HPI) is then calculated as follows;

$$HPI = \frac{{Q_{i} \times W_{i} }}{1}$$

where Q_i_ is the sub index of ith parameter, W_i_ is the unit weightage for ith parameter.

The critical pollution index value is 100. For the present study the *S*_*i*_ value was taken from the Standard Organization of Nigeria drinking water specifications standard (SON 2007).

### Metal Index

Another index used is the general Metal Index (MI) for drinking water (Bakan et al. 2010) which takes into account possible summation effect of heavy metals on the public health that help to quickly estimate the overall quality of drinking waters. Metal Index is given by the expression proposed by Caerio et al. (2005).

$$MI = \sum {[C_{i} /(MAC)_{i} } ]$$

where MAC is maximum allowable concentration and C_i_ is mean concentration of each metal. The higher the concentration of a metal compared to its respective MAC value the worse the quality of water. MI value > 1 is a threshold of warning (Bakan et al. 2010). The suitability of drinking water and it quality can be examined by determining its Metal Pollution Index as reported of previous works by Mohan et al. (1996), Prasad and Sangita (2008) and Singh (2008).

From the computed values on Table 5, the mean concentration of Heavy Metal Pollution Index was found to be 518.55 which were far above the critical threshold value of 100. Consequently, the result of this HPI value indicates that dugs well from scrap yard neighbourhood were critically polluted with heavy metals. Meanwhile ODS-5 location has its highest HPI value as 537.16. The presence of these Zn, Ni, Cd, Cu, and Pb found within this location were attributed from anthropogenic origin which includes industrial activities, traffic sources municipal sewage, domestic wastes, and atmospheric depositions. This find was in line with Manoj et al. (2012) that documented that metal from scrap yard, chemical weathering of minerals, as well as industrial discharges increase heavy metal concentration in water body.

**Table 5:** Mean value of HPI and MI recorded at different sampling locations

Sampling locations	HPI	MI
ODS-1	426.23	7.54
ODS-2	410.37	5.99
ODS-3	534.16	7.82
ODS-4	424.12	6.19
ODS-5	537.16	11.05
ODS-6	337.0	4.94
ODS-7	318.66	4.66
ODS-8	337.0	4.92
ODS-9	300.32	4.38
ODS-10	197.16	2.88
LFS-1	146.72	2.16
LFS-2	229.25	3.37
LFS-3	116.94	1.97
LFS-4	252.20	3.93
LFS-5	32.11	0.71
∑HPI = 518.55 ∑MI = 4.83		

The low computed HPI value of 32.11 in location LFS-5 might be as a result of dilution affect due to percolation and infiltration of rain water as was opined by Reza and Singh (2010).

The Metal Index (MI) of the waters from the two sampling locations were found to be higher as stated by the classification of Lyulko et al. (2001), Caerio et al. (2005) of Water Quality classification table which is shown in Table 4. The highest value 11.05 was recorded in sample location ODS-5 and was classified as strongly affected while the lowest value 0.71 in sample location LFS-5 is classified as pure which can be use for domestic and agricultural purposes.

### Potential Ecological Risk Index

The Potential Ecological Risk Index method was proposed by Hakanson (1980) from a sedimentology perspective to assess the characteristics and environmental behaviour of heavy metal contaminants. In further consideration of the heavy metals, the Hakanson method assesses their potential ecological and environmental effects with toxicology. The method comprises a single contamination coefficient, the toxic response factor for heavy metals, a comprehensive contamination measure as well as a Potential Ecological Risk Index.

The specific calculating formulas are as follows:

The single contamination coefficient is given by; $C_{f}^{i} = C_{sl}^{i} /C_{n}^{i}$, where $C_{f}^{i}$ is the contamination coefficient of a particular heavy metal, $C_{sl}^{i}$ is the measured data of heavy metals, and $C_{n}^{i}$ is the reference value. The comprehensive contamination measure (*C*_*d*_) is given by $C_{d} = \sum C_{f}^{i}$.

The Potential Ecological Risk Index ($E_{r}^{i}$) of a particular heavy metal equals to $E_{r}^{i} = T_{r}^{i} \times C_{f}^{i}$, where $T_{r}^{i}$ is the toxic response factor. The Potential Ecological Risk Index (RI) is given by ${\mathbf{RI}} = \sum E_{r}^{i}$.

The corresponding degrees of contamination and the evaluation standards for the levels of potential ecological risk in based on relevant studies (Dong et al. 2007; Jiao et al. 2012) and are shown in Table 6.

**Table 6:** Degree of contamination for particular heavy metals and the corresponding evaluation standards for potential ecological risk

$C^{i}_{f}$	<1, non-contamination	≥ 1 < 2, light	≥2, <3, moderate	≥3, heavy
*C*_*d*_	<8, low	≥ 8 < 16, moderate	≥ 16 < 32, relatively high	≥32, very high
$E^{i}_{r}$	<40, low	≥ 40 < 80, moderate	≥ 80 < 100, strong	≥320, extremely high
RI	<150, low	≥ 150 < 300, moderate	≥ 300 < 600, strong	≥600, very strong

The computation as shown in Table 7, reveals the degree of water contamination at the study area ranged from 0.71 to 7.9, with an average value of 4.57, reflecting light contamination level on sample locations but heavy contamination as composite average of 4.57 (see Table 7). The order of contamination in magnitude of the 5 heavy metals is, Cd > Pb > Zn > Cu > Ni while that of contamination at the sampling points is ODS-3 > ODS-1 > ODS-5 > ODS-4 > ODS-2 > ODS-6 > ODS-8 > ODS-7 > ODS-9 > LFS-4 > LFS-2 > ODS-10 > LFS-1 > LFS-3 > LFS-5.

**Table 7:** Computed contaminate level of the five heavy metals

Sample code	Zn	Ni	Cd	Cu	Pb	$\sum {\mathbf{C}}_{f}^{i}$	Contamination level
ODS-1	0.03	–	5.6	–	1.88	7.51	Light
ODS-2	0.02	–	6.0	–	–	6.02	Light
ODS-3	0.05	–	7.8	–	–	7.9	Light
ODS-4	0.03	–	6.2	–	–	6.23	Light
ODS-5	0.03	–	6.4	–	4.59	6.49	Light
ODS-6	0.04	–	4.9	–	–	4.94	Light
ODS-7	0.03	–	4.63	–	–	4.66	Light
ODS-8	0.02	–	4.9	–	–	4.92	Light
ODS-9	0.02	–	4.4	–	–	4.42	Light
ODS-10	0.02	–	2.87	–	–	2.8	Light
LFS-1	0.03	–	2.13	–	–	2.16	Light
LFS-2	0.03	–	3.33	–	–	3.36	Light
LFS-3	0.24	–	1.7	0.03	–	1.97	Light
LFS-4	0.26	–	3.67	–	–	3.93	Light
LFS-5	0.24	–	0.47	–	–	0.71	Light
Average value	0.073	–	4.33	0.03	3.24	4.57	Light
Contamination degree	NC	–	Heavy	NC	Heavy	Heavy	

The average value of the 5 heavy metals contamination coefficient was between 0.03 and 4.33 indicating high level of contamination. Cd had the largest contamination coefficient, especially in sample locations ODS-3 and ODS-5 with values 7.8 and 6.4 respectively indicating severe contamination. Therefore, targeted pollution control and management measures are urgently required to prevent the increase of Cd contamination and to limit potential harm.

**Table 8:** Ecological risk factor and Potential Ecological Risk Index of heavy metals in the study area

Sample code	Zn	Ni	Cd	Cu	Pb	$\sum E_{r}^{i}$	Risk grade
ODS-1	0.03	–	168	–	9.4	177.43	Moderate
ODS-2	0.02	–	180	–	–	180.02	Moderate
ODS-3	0.05	–	234	–	–	234.05	Moderate
ODS-4	0.03	–	186	–	–	186.03	Moderate
ODS-5	0.03	–	192	–	22.95	214.98	Moderate
ODS-6	0.04	–	147	–	–	147.04	Light
ODS-7	0.03	–	138.9	–	–	138.93	Light
ODS-8	0.02	–	147	–	–	147.02	Light
ODS-9	0.02	–	132	–	–	132.02	Light
ODS-10	0.02	–	86.1	–	–	86.12	Light
LFS-1	0.03	–	63.9	–	–	63.93	Light
LFS-2	0.03	–	99.9	–	–	99.93	Light
LFS-3	0.24	–	51	0.15	–	51.39	Light
LFS-4	0.26	–	110.1	–	–	110.36	Light
LFS-5	0.24	–	14.1	–	–	14.34	Light
Average value	0.073	–	130	0.15	16.8	132.24	
Potential ecological risk	Light	–	Very strong	Light	Light	Very strong	

Table 8 accounts for the Potential Ecological Risk Index of the heavy metals in the water at the study area based on Hakanson index method calculation and its division standard. The risk coefficients in most of the water samples were slight. The risk coefficients of the water in the ODS-1, ODS-2, ODS-3, ODS-4, and ODS-5 were moderate. In respect to the 5 heavy metals, the level of Cd contamination also posed the most serious potential ecological risk, with an index ranged of 14.1 and 234.

The risk coefficient of each water sample was either light or above. The risk coefficients of some of the water samples were high, although the mean value was computed as 130, which indicates a very strong risk. Cd pollution was the major problem in the study areas, with its overall content indicating a moderate degree of contamination. In other words, it calls for concern and deliberate steps must be taken in combating the effect and bio concentration and effective control of Cd emissions where necessary. The risk coefficients of Pb, Ni, Cu, and Zn at each site were all low, indicating that these other four heavy metals had a limited environmental impact at Owode-Onirin scrap yard and Lafenwa scrap yard as detected from the study.

## Conclusion

This study showed the potential discharge of various hazardous heavy metals in the water body through seepage in Owode-Onirin, Ikorodu area of Lagos Sate and Lafenwa scrap yard, Abeokuta, Ogun State due to uncontrolled activities of scrap yards. The results showed that the physicochemical parameters and of heavy metals concentration in surface water from most of sampling locations exceeded permissible limits set by Standard Organization of Nigeria drinking water specifications standard (SON 2007) and WHO (2004) for drinking water. Variations in concentrations of these various heavy metals in water sampled are a consequence of a wide range of scrap yard activities within the neighbourhood.

The overall HPI calculated based on the mean concentration of the heavy metals was computed to be 518.55 which is more than the critical threshold pollution index value of 100, indicating that the selected water samples from the scrap yard neighborhood were critically contaminated with heavy metals. The result of the MI also computed to be 4.83 suggests that the selected water samples were seriously polluted with heavy metals. The study further revealed the impact of anthropogenic sources on the pollution load of the water in scrap yard neighbourhood.

The Hakanson Potential Ecological Risk Index was also used to analyze and compute the concentration of Cd, Pb, Cu, Zn, and Ni in water samples from neighbourhood of scrap yards, and the levels of contamination and the potential ecological toxicity of the heavy metals in the water samples were obtained. The Hakanson Potential Ecological Risk Index indicated that the overall pollution in the water samples at the two locations were moderate. The risk coefficient of Zinc, Lead, Cupper and Nickel all had light levels of contamination while the level of Cadmium contamination posed the most serious potential ecological risk, with an index value ranged between 14.1 and 234. Heavy metals contamination in order of magnitude were Cd > Pb > Zn > Cu > Ni. Cadmium (Cd) had the highest toxic response factor of 30 and represented the greatest ecological hazard. The study concluded proactive and concrete measures must be taken to reduce and control the heavy metals contamination in surface water within the scrap yard neighbourhood, more specifically with respect to Cadmium.
